# Clinical Use of DPP-4 Inhibitors

**DOI:** 10.3389/fendo.2019.00389

**Published:** 2019-06-19

**Authors:** Baptist Gallwitz

**Affiliations:** Divsion for Diabetes, Endocrinology and Nephrology, Department of Internal Medicine, Tübingen University Hospital, Tübingen, Germany

**Keywords:** type 2 diabetes, DPP-4 inhibitors, oral antidiabetic, incretin-based therapy, gliptin, combination therapy

## Abstract

DPP-4 inhibitors were introduced for the treatment of type 2 diabetes in 2006. They stimulate insulin secretion and inhibit glucagon secretion by elevating endogenous GLP-1 concentrations without an intrinsic hypoglycaemia risk. Their efficacy potential to lower HbA1c is in the range between 0.5 and 1.0% and their safety profile is favorable. DPP-4 inhibitors are body weight neutral and they have demonstrated cardiovascular safety. Most compounds can be used in impaired renal function. Guidelines suggest the additional use of DPP-4 inhibitors after metformin failure in patients that do not require antidiabetic therapy with proven cardiovascular benefit. Recently, DPP-4 inhibitors have increasingly replaced sulfonylureas as second line therapy after metformin failure and many metformin/DPP-4 inhibitor fixed dose combinations are available. In later stages of type 2 diabetes, DPP-4 inhibitors are also recommended in the guidelines in triple therapies with metformin and SGLT-2 inhibitors or with metformin and insulin. A treatment with DPP-4 inhibitors should be stopped when GLP-1 receptor agonists are used. DPP-4 inhibitors can be used as monotherapy when metformin is contraindicated or not tolerated. Some studies have shown value of initial metformin-DPP-4 inhibitor combination therapy in special populations. This article gives an overview on the clinical use of DPP-4 inhibitors.

## Introduction

**Figure 1:**
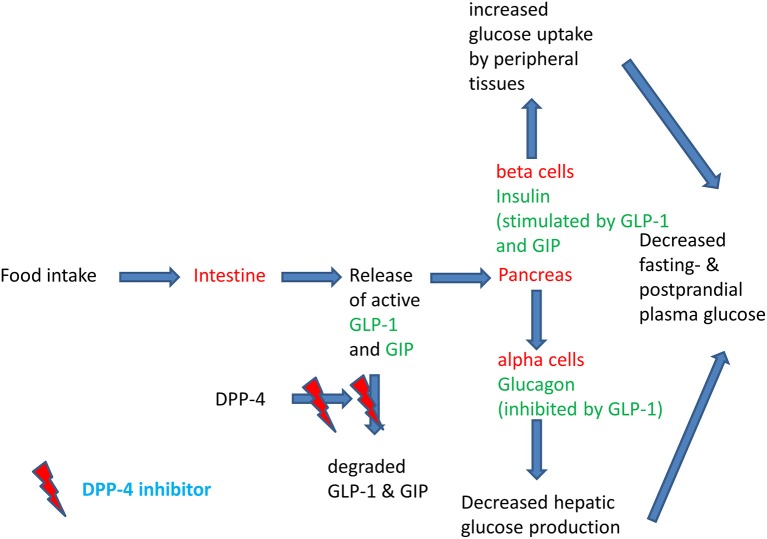
Physiology of the post-prandial regulation of glucose homoeostasis by the incretin system and the action of DPP-4 inhibitors. Modified after (11).

The regulation of insulin secretion is important to maintain euglycaemia. In type 2 diabetes, a deterioration of insulin secretion and the development of peripheral insulin resistance lead to the development of hyperglycaemia. Insulin is physiologically constantly secreted to a small extent during the fasting state in order to enhance glucose uptake by the peripheral tissues. After a meal, insulin secretion is stimulated quickly and considerably in order to maintain plasma glucose concentrations within a narrow physiological range (1). The post-prandial stimulation of glucose is not only promoted by the post-prandial rise in glucose concentrations, but also by the gastrointestinal hormones glucagon-like peptide-1 (GLP-1) and gastric inhibitory polypeptide (GIP). These two hormones stimulate insulin secretion under hyperglycemic conditions and contribute to ~70% of the post-prandial insulin secretion. They are called incretin hormones to highlight their important physiological action in stimulating post-prandial insulin secretion (2–4). The so-called incretin effect describes the phenomenon that orally ingested glucose leads to a much higher insulin response than intravenously administered glucose. In type 2 diabetes, the incretin effect is diminished and in parallel, post-prandial insulin secretion is deteriorating (2, 5). A pharmacological elevation of GLP-1 is able to restore insulin secretion in type 2 diabetes (6). Since the GLP-1 dependent stimulation of insulin secretion is only present under hyperglycaemic conditions, there is a very low intrinsic risk of hypoglycaemia. GLP-1 has another beneficial effect in type 2 diabetes that contributes to maintaining euglycaemia: in type 2 diabetes glucagon secretion is excessively stimulated and glucagon stimulates hepatic glucose production. GLP-1 inhibits glucagon secretion under hyperglycaemic conditions and thereby improves glycaemia. GLP-1 is a peptide hormone with a short plasma half-life of a few minutes (4, 7). The short biological half-life is due to a rapid enzymatic degradation of GLP-1 (and GIP also) by the enzyme dipeptidyl peptidase IV (DPP-4) (8). DPP-4 can be inhibited by orally active small molecules, the DPP-4 inhibitors. The administration of DPP-4 inhibitors leads to a 2-3-fold elevation of endogenous GLP-1 concentration (9). GLP-1 is a substrate with a high affinity for DPP-4 (“direct target”). Besides GLP-1, DPP-4 also has other substrates and their elevation by DPP-4 inhibition can also contribute to a normalization of glycaemia in type 2 diabetes (“indirect target” or “off-target”) (10). Figure 1 highlights the physiology of the incretin hormones after a meal and the mode of action of DPP-4 inhibitors (7).

DPP-4 inhibitors have become an increasingly firmly established class of oral antidiabetic agents for the treatment of type 2 diabetes. Sitagliptin was the first agent introduced (in 2006) with other substances having followed soon after. The most widely substances used are sitagliptin, linagliptin, vildagliptin, saxagliptin, and allogliptin. Anagliptin, gemigliptin, and teneligliptin are used in Asian countries. DPP-4 inhibitors are implemented into the treatment algorithms of type 2 diabetes in many national and international guidelines (12).

**Figure 2:**
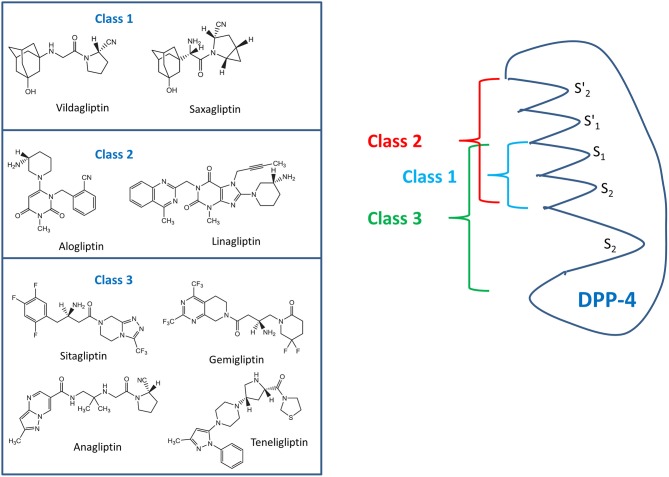
Classes of DPP-4 inhibitors with the various commonly used DPP-4 inhibitors (left side) and the binding domains of the various classes to specific areas of the DPP-4 molecule (right side) according to Tomovic et al. (13) and Nabeno et al. (14).

The various DPP-4 inhibitors do not form a homogenous class of molecules, and they show different interactions with the active site of the enzyme molecule. The binding modes of the clinically most widely used DPP-4 inhibitors have been characterized and based on these findings, three different classes of DPP-4 inhibitors have been proposed: Class 1 comprises of saxagliptin and vildagliptin, interacting with the S1- and S2 subsites of the active center and covalently binding with Ser630 of the DPP-4 molecule. Alogliptin and linagliptin also bind to S1 and S2 but also interact with S1' and/or S2' and belong to class 2. Sitagliptin, anagliptin, gemigliptin, and teneligliptin form class 3 of the DPP-4 inhibitors (see Figure 2) (13, 14). The mentioned DPP-4 inhibitors are orally active, rapidly absorbed, and suitable for once daily or twice daily administration, leading to a DPP-4 inhibition of 70–90% over 24 h. Except for linagliptin, they are eliminated renally after little metabolization. Saxagliptin is metabolized generating an active metabolite. Linagliptin is eliminated via a biliary route (10, 13, 15, 16).

The clinically most relevant and important action of DPP-4 inhibitors is the endogenous elevation of the incretin hormone concentration of GLP-1 that consecutively leads to a glucose-dependent stimulation of insulin secretion and an inhibition of glucagon secretion (10). The insulinotropic effect of DPP-4 inhibitors explains the phenomenon that this class increasingly is replacing the use of sulfonylureas as insulin releasing agents; especially since the intrinsic hypoglycaemia risk of DPP-4 inhibitors is very low. Apart from that, DPP-4 inhibitors are body weight neutral, whereas sulfonylurea therapy is associated with body weight gain (17, 18). The approved DPP-4 inhibitors demonstrate a high specificity toward DPP-4 and do not inhibit other peptidases. All DPP-4 inhibitors can be given in a standard dose without the need for dose titrations (10). GLP-1 has the highest substrate specificity for DPP-4, but DPP-4 has additional substrates and inhibition therefore extends the biological half-life of the incretin hormone GIP, brain natriuretic peptide, and many others (10, 19).

This article gives an overview on the characteristics of the various DPP-4 inhibitors in clinical use and highlights the positioning of the drug class in the guidelines for the treatment of type 2 diabetes.

## DPP-4 Inhibitors and Their Clinical Characteristics

The DPP-4 inhibitors available demonstrate a high efficacy in inhibiting DPP-4, and under clinical conditions DPP-4 is inhibited by >80–90%. This inhibition consecutively leads to post-prandial GLP-1 plasma concentrations that are elevated 2-3-fold and mediates the glucose-dependent stimulation of insulin secretion and inhibition of glucagon secretion (10, 19). In addition to this “endocrine” action, the local inhibitory effect of DPP-4 inhibitors on GLP-1 degradation in the intestinal mucosa may contribute to favorable metabolic regulation by stimulating the autonomic afferent nervous system (10, 19). The bio-availability of the DPP-4 inhibitors is very good and the pharmacodynamic- as well as the pharmacokinetic characteristics lead to the above mentioned clinically sufficient DPP-4 inhibition with once daily dosing (only vildagliptin has to be dosed twice daily) (18, 19). Omarigliptin is a long-acting DPP-4 inhibitor for once weekly dosing that is presently approved in Japan, but not in Europe or American countries (20). In the clinical development programs and later in broad use after approval, no drug-drug interactions of DPP-4 inhibitors with other antidiabetic drugs or with common medications like antihypertensives, lipid lowering agents, diuretics or anticoagulants were observed (12, 19, 21, 22). DPP-4 inhibitors are capable of lowering HbA1c percentage by ~0.5–1 unit. The HbA1c reduction, as with other antihyperglycaemic agents depends largely on the patient population studied, the baseline glycaemic situation at the beginning of the observation and the concomitant therapy, including life-style intervention (12, 19, 21, 22). In the phase III clinical developmental programs of the DPP-4 inhibitors, non-inferiority was demonstrated after 1 and 2 years for these substances compared to sulfonylureas with respect to the glycaemic parameters HbA1c as well as fasting- and post-prandial plasma glucose concentrations. Similar efficacy of DPP-4 inhibitors to either metformin or pioglitazone was also shown in formerly drug-naïve patients who did not reach glycaemic goals with non-pharmacological interventions. DPP-4 inhibitors are body weight neutral and they are generally tolerated well without side effects (see also corresponding section below) (12, 19, 21, 22). Table 1 gives a summary of the clinical phase III studies with at least 52 weeks duration on the efficacy of DPP-4 inhibitors compared to sulfonylureas as add on therapy to metformin, respectively, in patients not reaching their glycaemic goals with a monotherapy of metformin.

**Table 1:** Efficacy of DPP-4 inhibitors in the clinical phase III programmes with at least 52 weeks duration as add on therapy to metformin compared to sulfonylureas.

**Substance/study** **[References]**	**Observation time** **[weeks]**	**Intervention**	**Change in HbA1c** **[%]**	***n* [pat.]**	**HbA1c <7.0%** **[% pat.]**	**Change in body weight** **[kg]**	**Hypoglycaemic episodes** **[% pat.]**
Alogliptin Del Prato (23)	104	Alogliptin 12.5 mg Alogliptin 25 mg Glipizide >5 mg	−0.68 −0.72 −0.59	874	45.6* 48.5** 42.8	−0.68 −0.89 +0.95	1.4 1.4 23.2
Linagliptin Gallwitz (24)	104	Linagliptin 5 mg Glimepiride >1 mg	−0.16 −0.36	1551	30.0* 35.0	−1.4 +1.3	7.0 36.0
Saxagliptin Göke (25)	104	Saxagliptin 5 mg Glipizide 5–20 mg	−0.41 −0.35	858	23.1 22.7	−1.5 +1.3	3.5 38.4
Sitagliptin Seck (26)	104	Sitagliptin 100 mg Glipizide 5–20 mg	−0.54 −0.51	1172	63.0 59.0	−1.6 +0.7	5.0 34.0
Vildagliptin Filozof (27)	52	Vildagliptin 2 × 50 mg Gliclazide <320 mg	−0.81 −0.85	1007	30.0* 32.0	+0.08 +1.36	n.a.
Vildagliptin Matthews (28)	104	Vildagliptin 2 × 50 mg Glimepiride <6 mg	−0.10 −0.10	3118	37.0 38.0	−0.3 +1.2	2.0 18.0

Alogliptin (23) *p < 0.001 vs. sulfonylurea (SU) (non inferiority shown), **p < 0.010 vs. SU (superiority shown). Linagliptin (24) *p < 0.05 vs. SU (completers, two-sided). Vildagliptin (27) *p = 0.257 vs. SU (non inferiority shown).

The most important and most frequently used indication for DPP-4 inhibitors is their add-on use in patients who are not sufficiently controlled on metformin monotherapy (12, 17–19, 21, 22). Under these circumstances and this indication, a significantly higher percentage of patients reaches the glycaemic goal HbA1c <7.0% without hypoglycaemia and without body weight gain compared to a treatment with a metformin plus sulfonylurea combination and the glycaemic efficacy of the DPP-4 inhibitors is non-inferior to sulfonylurea (18, 19, 21, 24–30). Several fixed dose combinations of DPP-4 inhibitors with metformin are available and may safely be used in patients on this treatment combination, especially, when a reduction in the daily tablet load is important. DPP-4 inhibitors can on the other hand also be combined with an oral therapy with a SGLT-2 inhibitor (in dual combination when metformin is contraindicated or not tolerated or in triple therapy with metformin). Regarding the combination with injectable antidiabetic treatment, DPP-4 inhibitors can be used together with insulin. They have shown a further reduction of HbA1c percentage between 0.5 and 0.7 in this combination and in some studies a lower rate of hypoglycaemic events was observed (12, 18, 21, 31).

DPP-4 inhibitors can be administered in patients with impaired kidney function due to the good safety and tolerability. Except for linagliptin, which is eliminated via a biliary route, all other DPP-4 inhibitors are excreted in the urine (22, 29, 32, 33). Dose adjustments have to be observed for the various DPP-4 inhibitors with renal elimination in dependence of the compound used and the severity of renal impairment (21, 22, 29, 32).

DPP-4 inhibitors are contraindicated in type 1 diabetes as well as during pregnancy and lactation.

## Extraglycaemic Effects on Lipids

Monami et al. performed a metanalysis comparing the extraglycaemic effects of various antihyperglycaemic agents on the lipid profile in type 2 diabetes (34). In this analysis, DPP-4 inhibitors lowered total cholesterol and triglycerides (34). In comparison to other antihyperglycaemic agents, DPP-4 inhibitors lowered total cholesterol slightly more than other agents (total cholesterol −0.18 [−0.29; −0.06] mmol/L/−7.0 [−11.2; −2.50] mg/dL; *p* = 0.002) (35). The clinical significance of this slight difference is debatable, but the effect is mentioned here as a possible “off-target” effect of DPP-4 inhibitors.

In smaller acute metabolic studies with DPP-4 inhibitors, a reduction of the post-prandial rise in triglycerides, and apolipoprotein-B 48 was observed. This effect may be explained by a DPP-4 inhibitor-dependent reduction in intestinal lipoprotein production and consecutive lowering of circulating chylomicrons (36, 37).

## Side Effects of DPP-4 Inhibitors

DPP-4 inhibitors have shown good safety- and tolerability profiles in the phase III clinical study programs and the most frequent adverse events observed were nasopharyngitis and skin lesions. In most studies, the adverse events did not lead to treatment discontinuation (12, 19, 21, 22, 24–33). The efficacy and safety profile of the DPP-4 inhibitors shows a favorable profile of the DPP-4 inhibitors especially for patients with renal impairment as well as elderly subjects with type-2-diabetes. In clinical use monitored by post-marketing surveillance and in the long-term cardiovascular safety studies, no serious imbalances in safety signals were observed (12, 19, 21, 22, 24–33).

An intensive discussion on the pancreatic safety of incretin-based therapies initiated by publications of a single group have led to a thorough evaluation of non-clinical and clinical data by the European Medicines Agency (EMA) and the US Food and Drug Administration (FDA) (38–45). This extensive evaluation did not find a causal relationship between a therapy with incretin-based therapies and pancreatic safety. Patients with type 2 diabetes have an approximately two-fold risk for acute pancreatitis and a label indicating an acute pancreatitis risk has been added to all DPP-4 inhibitors. Several retrospective studies and meta-analyses on the association of DPP-4 inhibitor therapy and pancreatitis have altogether shown a low risk for acute pancreatitis (46, 47). Additionally, the large cardiovascular safety studies did not show a significant respective signal. A recent analysis calculated an estimated number needed to harm of 1,066 associated with a DPP-4 inhibitor therapy (12, 47–49). A recent review on the potential association between DPP-4 inhibitor use and cancer did not reveal an increased cancer risk including pancreatic cancer (12, 50).

Regarding skin lesions, bullous pemphigoid, a rare autoimmune skin disease, was found to be associated with the use of DPP-4 inhibitors in a retrospective analysis of more than 9,000 patients treated in Japan during the years 2009–2017. The prevalence of bullous pemphigoid was 0.0859% in total, with a trend toward a higher risk associated with vildagliptin use compared to the other DPP-4 inhibitors (51). The EMA and FDA imposed a respective label (52, 53). Data from the large cardiovascular safety studies with DPP-4 inhibitors (see section below) did not show a respective signal. Here, the incidence rate in studies was very low and the study data have shown different results, so that further research is warranted on this observation. Pathophysiologically, the skin lesions could only be explained as “indirect target” effect of DPP-4 inhibitors.

In total, however, the DPP-4 inhibitor class has demonstrated a good safety and tolerability spectrum that justifies the wide use of the class.

## Cardiovascular Safety Studies With DPP-4 Inhibitors

The “Clinical Guidance for Pharmaceutical Industry–Diabetes Mellitus—Evaluating Cardiovascular Risk in New Antidiabetic Therapies to Treat Type 2 Diabetes” established by the FDA in 2008 as a consequence of adverse safety signals of rosiglitazone has set up the requirement for novel diabetes medications to prove cardiovascular safety of these substances in comparison to standard therapy under glycaemic equipoise. The study design of these studies primarily aims at demonstrating non-inferiority (safety) of a novel substance compared to a control arm with standard of care excluding the novel drug. In order to rule out direct glucose effects on the macro- and microvascular event rate, glucose parameters are kept as closely overlapping as possible (54–56). Most of these studies have a combined predefined endpoint of the classical MACE-3 (major adverse cardiovascular endpoint) consisting of cardiovascular death, non-fatal myocardial infarct and non-fatal stroke. The studies are event driven and powered to proof non-inferiority. In case non-inferiority is reached at the end of the study, a statistical testing for superiority (cardiovascular benefit of the tested novel substance) can be performed. The non-inferiority design requires a smaller study participant sample and a shorter observation period. In most studies, a study population with pre-existing cardiovascular disease is chosen in order to aim at a higher event rate in a shorter study duration (54–61).

So far four studies for cardiovascular safety studies have been completed and published: The EXAMINE (Examination of Cardiovascular Outcomes with Alogliptin vs. Standard of Care) study for alogliptin (58, 62), the Savor-TIMI-53 study (Saxagliptin Assessment of Vascular Outcomes Recorded in Patients with Diabetes Mellitus–Thrombolysis in Myocardial Infarction 53) for saxagliptin (60, 63), the TECOS study (Trial Evaluating Cardiovascular Outcomes With Sitagliptin) for sitagliptin (59, 64) and the CARMELINA study (Cardiovascular safety and Clinical Outcome with Linagliptin) for linagliptin (61, 65). All these studies have proven cardiovascular safety for the respective DPP-4 inhibitors. Comparing the effect on the primary endpoint, the studies showed a very homogenous result. Regarding the secondary endpoint hospitalization due to heart failure, the results of the studies are heterogenous. Saxagliptin therapy was associated with a significantly increased rate of hospitalization due to heart failure in comparison to standard therapy (3.5% vs. 2.8%; HR 1.27, 95% CI 1.07–1.51) (63). This imbalance did not affect the primary endpoint and the incidence rate was higher in patients with a previous history of heart failure and independent of renal function at study baseline (57, 66, 67). A similar, but not significant signal was observed for alogliptin (3.9 vs. 3.3%; HR 1.19, 95% CI 0.9–1.58), but not for the other DPP-4 inhibitors as in the TECOS study for sitagliptin (HR 1.00, 95% CI 0.83–1.20) or the CARMELINA study for linagliptin (HR 0.90, 95% CI 0.74–1.08) (57, 62, 64, 65, 68). The cardiovascular disease status of the patients differed in the studies and may have influenced the heart failure outcome (57). As a consequence, saxaglitpin treatment should be avoided in patients with heart failure. Table 2 gives an overview on the cardiovascular safety data of the DPP-4 inhibitors alogliptin, linagliptin, saxagliptin, and sitagliptin. The cardiovascular safety study CAROLINA (CARdiovascular Outcome Trial of LINAgliptin vs. Glimepiride in Type 2 Diabetes) with linagliptin comparing linagliptin treatment as add on to metformin directly with a therapy with the sulfonylurea glimepiride will bring results later in 2019 and may give additional insights into the association and mechanisms linking hypoglycaemic- with cardiovascular events (24, 69). In summary, the DPP-4 inhibitors have demonstrated cardiovascular safety in multiple studies. These results are the basis for a positioning of the DPP-4 inhibitors as second-line therapy for the treatment of type 2 diabetes, especially, when hypoglycaemia should strictly be avoided (see section below).

**Table 2:** Cardiovascular outcome studies with DPP-4 inhibitors; baseline characteristics of patients; and primary MACE endpoint.

**Study [References]/DPP-4 inhibitor**	**EXAMINE (58, 62) Alogliptin**	**SAVOR-TIMI 53 (60, 63) Saxagliptin**	**TECOS (59, 64) Sitagliptin**	**CARMELINA (61, 65) Linagliptin**	**CAROLINA (69) Linagliptin**
Number (*n*)	5,400	16,492	14,671	6,979	6,103 (enrolled)
Primary endpoint/MACE hazard ratio (95% CI)	0.96 (n.a., 1.16)	1.00 (0.89, 1.12)	0.99 (0.89, 1.10)	1.02 (0.89, 1.17)	Results expected June 2019
Secondary endpoint hospitalization for heart failure (95% CI)	1.19 (0.89, 1.59)	1.27 (1.07, 1.51)	1.00 (0.83, 1.20)	0.90 (0.74, 1.08)	
Comparator	Placebo	Placebo	Placebo	Placebo	Glimepiride
follow up time (years)	1.5	2.1	3.0	2.3	
**BASELINE CHARACTERISTICS**					
History of CVD (%)	100 (ACS was inclusion criterion)	78	100	57	34
Type of CVD	ACS <90 days	≥40 y + CV disease (CHD, CVD, PVD) or >55 y + ≥1 CV risk factor	CHD, CVD, PVD	CVD (57%), CKD (74%), both (33%)	CVD (34.5%)
Age (years)	61	65	66	66	64
HbA1c (%)	8.0	8.0	7.2	7.9	7.2
Diabetes duration (years)	7.2	10	9.4	15	6
eGFR <60 ml/min/1.73 kg/m^2^ (%)	29	16	9 (<50 ml/min/1.73 kg/m^2^)	62	18
BMI (kg/m^2^)	28.7	31	30.2	31.3	30
Insulin treatment (%)	30	41	23	58	0
Statin treatment (%)	90	78	80	72	64
ACEI/ARB treatment (%)	82	82	82	81	75
Aspirin treatment (%)	91	75	79	68	50

*ACEI, angiotensin converting enzyme inhibitor; ARB, angiotensin receptor blocker; ACS, acute coronary syndrome; BMI, body mass index: CVD, cardiovascular disease; CHD, chronic heart disease; CKD, chronic kidney disease; PVD, peripheral vascular disease*.

## Positioning of DPP-4 Inhibitors in the Treatment Algorithm of type 2 Diabetes

In 2018 the American Diabetes Association (ADA) and the European Association for the Study of Diabetes (EASD) published a new joint position statement for the treatment of type 2 diabetes (70, 71). The ADA has adopted these recommendations in their annual recommendations “Standards of Medical Care in Diabetes” in 2019 (72). The treatment algorithm recommends a patient centered and individualized treatment approach with the goal to prevent diabetes-related complications and to optimize quality of life in patients with type 2 diabetes. As in the previous recommendations by the ADA and EASD (73, 74), life-style intervention (with patient education and motivation, increase of physical activity, and healthy eating) is still in the beginning and center of therapy, followed by pharmacological therapy with metformin (70–74).

If therapeutic goals are not met with these measures, certain patient characteristics determine the further recommended treatment options. Patients with established cardiovascular disease should receive a pharmacological treatment intensification with an agent that has demonstrated benefit in cardiovascular safety studies: In patients with pre-existing atherosclerotic cardiovascular disease (ASCVD), a GLP-1 receptor agonist, or SGLT-2 inhibitor with characterized cardiovascular safety should be used. Patients in whom heart failure (HF) or chronic kidney disease (CKD) is prevalent, should receive a treatment intensification with an SGLT-2 inhibitor with the respective evidence from clinical studies due to the respective data (70–72). In both patient populations with pre-existing cardiovascular disease, DPP-4 inhibitors are recommended as one possible third-line therapy besides SGLT-2 inhibitors, thiazolidinediones, or insulin, when therapeutic goals are not met with the previous dual combination. The combination of GLP-1 receptor agonists and DPP-4 inhibitors is not recommended, not due to safety issues, but since a clinically significant additional benefit is not expected (see below) (70–72). In patients with pre-existing HF, an exception is noted for saxagliptin. This DPP-4 inhibitor is contraindicated in this patient group on the basis of the respective cardiovascular safety study that has shown a significant increase of hospitalization for heart failure as secondary endpoint in the saxagliptin arm (63, 66).

The suggested treatment algorithm of the ADA and EASD stratifies patients without pre-existing cardiovascular disease further. In patients in whom the major therapeutic goal is the compelling need to minimize the therapeutic risk pf hypoglycaemia, DPP-4 inhibitors are recommended as second line therapy after metformin failure at the same level as second line treatment options with GLP-1 receptor agonists, SGLT-2 inhibitors, or TZDs. The choice of the agent depends on the patient characteristics (e.g., kidney function), treatment preference (oral therapy vs. injectable therapy) and comorbidities/co-medication (70–74). The treatment option with the combination of a DPP-4 inhibitor as add on to metformin is currently the most widely used. There are a number of reasons for the frequent use of this therapy: from a pathophysiological point, the metformin/DPP-4 combination leads to higher GLP-1 concentrations compared to a therapy with a DPP-4 inhibitor alone (75). A historical reason is that DPP-4 inhibitors were introduced in 2006 and therefore, the practical experience with this class of agents is deeper and more widespread than that with SGLT-2 inhibitors. The safety profile of the DPP-4 inhibitors is also favorable and last not least, there are fixed dose combinations with metformin and DPP-4 inhibitors available. In case patients have a dual therapy with either a SGLT-2 inhibitor or a TZD and are not at goal, a further treatment escalation to a triple therapy with a DPP-4 inhibitor can be implemented (70–72). Patients with a dual therapy with metformin and a DPP-4 inhibitor can also receive a further treatment intensification with a SGLT-2 inhibitor or a TZD (70–74).

The triple therapy of metformin, a DPP-4 inhibitor and a SGLT-2 inhibitor has a very low treatment related hypoglycaemia risk, leads to a further, almost additive reduction of glycaemic parameters, a body weight loss in the same range as with a mono- or dual therapy with a SGLT-2 inhibitor and also to the expected SGLT-2 inhibitor induced reduction of blood pressure. All three substances can be given in standard doses (76–85). Even though there are studies showing an effective and safe reduction of glycaemic parameters with initial combination therapies with DPP-4 inhibitors, initial combinations are not generally recommended in the new treatment recommendations (70–72, 76–85).

Another patient segment noted in the recommendations is the group with a foremost treatment goal to minimize body weight gain and to promote weight loss. Here, after metformin failure, GLP-1 receptor agonists, or SGLT-2 inhibitors are recommended as second line therapy or as third line therapy in combination. DPP-4 inhibitors may be placed in this patient group in those individuals who do not tolerate a treatment with GLP-1 receptor agonists (70–72). There is a strong recommendation not to combine DPP-4 inhibitors with GLP-1 receptor agonists, and DPP-4 inhibitor therapy should be discontinued when treatment is escalated with an injectable therapy at later stages with a GLP-1 receptor agonist (70–72).

DPP-4 inhibitors can also be combined with an insulin therapy. Some studies have shown a reduction of the insulin dose and in hypoglycaemic episodes. For patients who are not well-controlled on a treatment with metformin and basal insulin, a treatment intensification with a DPP-4 inhibitor may be feasible. This is a less complicated treatment intensification than adding a short-acting mealtime insulin. The basal insulin/DPP-4 inhibitor combination is a practical and a less complicated treatment option without the need for multiple injections during the day, less need for glucose self-measurements and dose adjustments for insulin (70–74).

**Figure 3:**
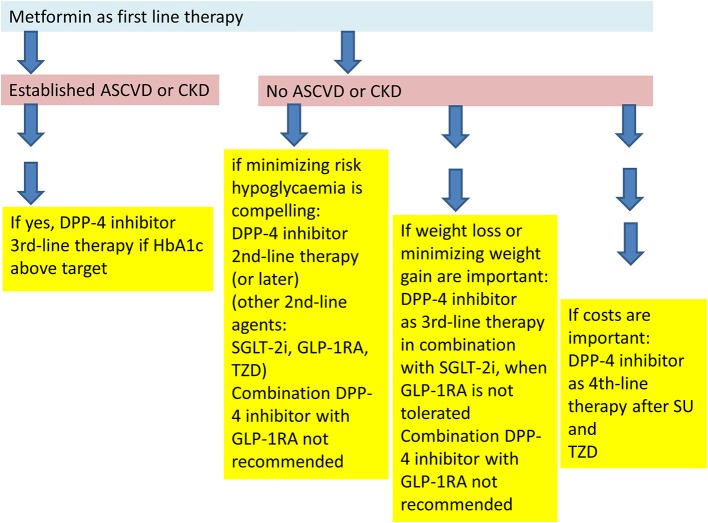
Placement of DPP-4 inhibitors into the treatment algorithm according to the recommendations of the American (ADA)- and European (EASD) Diabetes Associations (70, 71).

The treatment algorithm recommended by ADA and EASD is summarized in Figure 3 (72, 73).

## Conclusions

DPP-4 inhibitors are important oral antidiabetic agents that are placed as second-line therapy after metformin failure as insulinotropic agents that have no intrinsic hypoglycaemia risk and are body weight neutral. Under hyperglycaemic conditions they additionally inhibit glucagon secretion. They should mainly be used as second-line therapy as add on to metformin in patients with type 2 diabetes who have no pre-existing cardiovascular disease and have a therapeutic goal to avoid hypoglycaemic events. Fixed dose combinations with metformin are widely used. The side effect profile of DPP-4 inhibitors is favorable, there are few treatment-limiting adverse effects and DPP-4 inhibitors have shown cardiovascular safety. Another favorable characteristic of the DPP-4 inhibitors is their efficacy and safety profile in patients with impaired renal function. Apart from the above mentioned indication and placement, DPP-4 inhibitors can also be administered in triple combination treatment with either metformin and SGLT-2 inhibitors or with metformin and insulin. In combination with insulin, some studies have shown a reduction in hypoglycaemic episodes due to a reduction in the insulin dose. A combination with GLP-1 receptor agonists is not recommended since DPP-4 inhibitors as well as GLP-1 receptor agonists elevate “GLP-1” plasma concentrations. DPP-4 inhibitors increase the endogenous GLP-1 concentrations ~2-3-fold, GLP-1 receptor agonists lead to 8-10-fold concentrations. An exploratory study with sitagliptin and the GLP-1 receptor agonist liraglutide did not show additive effects, and whether there may be additional effects using shorter acting GLP-1 receptor agonists and DPP-4 inhibitors has not been studied yet (70–72, 86). DPP-4 inhibitors are increasingly replacing sulfonylureas as insulinotropic agents, but they can frequently also be a good therapeutic alternative to other treatment options such as glitazones or glucosidase inhibitors.

## Author Contributions

The author confirms being the sole contributor of this work and has approved it for publication.

### Conflict of Interest Statement

BG has served as a consultant for AstraZeneca, Boehringer Ingelheim, Bristol-Myers Squibb, Merck (MSD) and Novartis as manufacturers of DPP-4 inhibitors and has received honoraria from these companies for lectures.
